# A Retrospective Global Assessment of Factors Associated With COVID-19 Policies and Health Outcomes

**DOI:** 10.3389/fpubh.2022.843445

**Published:** 2022-05-09

**Authors:** Angela Jeong Choi, Andrew C. Hean, Julia K. Lee, Nguyen D. Tran, Tracy Kuo Lin, Dorie E. Apollonio

**Affiliations:** ^1^School of Pharmacy, University of California, San Francisco, San Francisco, CA, United States; ^2^Department of Social and Behavioral Sciences, Institute for Health & Aging, University of California, San Francisco, San Francisco, CA, United States; ^3^Department of Clinical Pharmacy, University of California, San Francisco, San Francisco, CA, United States

**Keywords:** pandemics, COVID-19, SARS-CoV-2, health policy, health services, masks, social distancing, lockdowns

## Abstract

**Background:**

The 2019 Global Health Security (GHS) Index measured the capacities of countries to prepare for and respond to epidemics and pandemics. However, the COVID-19 pandemic revealed that GHS Index scores were poorly correlated with ability to respond to infectious disease threats. It is critical to understand how public health policies may reduce the negative impacts of pandemics.

**Objective:**

To identify non-pharmaceutical interventions (NPIs) that can minimize morbidity and mortality during the COVID-19 and future pandemics, this study examined associations between country characteristics, NPI public health policies, and COVID-19 outcomes during the first year of the pandemic, prior to the introduction of the COVID-19 vaccine. This global analysis describes worldwide trends in policy implementation and generates a stronger understanding of how NPIs contributed to improved health outcomes.

**Design:**

This cross-sectional, retrospective study relied on information drawn from publicly available datasets through December 31, 2020.

**Primary and Secondary Outcome Measures:**

We conducted multivariate regressions to examine associations between country characteristics and policies, and policies and health outcomes.

**Results:**

Countries with higher health service coverage prior to the pandemic implemented more policies and types of policies. Countries with more bordering countries implemented more border control policies (0.78^**^), and countries with denser populations implemented more masking policies (0.24^*^). Across all countries, fewer COVID-19 cases and deaths per million were associated with masking (−496.10^*^, −7.57), testing and tracing (−108.50^**^, −2.47^**^), and restriction of movement (−102.30^*^, −2.10^*^) policies, with stronger associations when these policies were mandatory rather than voluntary.

**Conclusions:**

Country characteristics, including health service coverage, number of bordering countries, and population density, may predict the frequency and nature of public health interventions. Countries with higher health service coverage may have the infrastructure to react more efficiently to a pandemic, leading them to implement a greater number of policies. Mandatory masking, testing and tracing, and restriction of movement policies were associated with more favorable COVID-19 population health outcomes. While these results are consistent with existing COVID-19 mathematical models, policy effectiveness depends on how well they are implemented. Our results suggest that social distancing policies were less effective in reducing infectious disease risk, which may reflect difficulties with enforcement and monitoring.

## Introduction

After the 2009 H1N1 pandemic, the World Health Organization (WHO) concluded that the world was unprepared for a future influenza outbreak or sustained public health crisis (1). During the 2009 pandemic only 10% of all states met the WHO guidelines (2). In November 2019, the Global Health Security Index attempted the first comprehensive assessment of capacity to avert infectious disease threats across 195 countries. It categorized countries as “most,” “more,” and “least” prepared in six categories including prevention, detection and reporting, rapid response, health system, compliance with international norms, and risk environment. However, in an actual pandemic the following year, these predictions were poorly correlated with COVID-19 response. For example, the U.S. earned the highest GHS score, yet in 2020 reported one of the highest numbers of COVID-19 cases per capita (3, 4).

By the end of 2020, the COVID-19 pandemic had caused over 1.8 million deaths and countries have had mixed success implementing interventions to address the pandemic (5–8). The limited evidence on the relationship between a country's expected preparedness and health outcomes demonstrates the need to identify factors associated with an effective pandemic response. The recent 2009 H1N1 pandemic was estimated to have mortality of up to 575,500 people worldwide in the first year of the virus's circulation (9), demonstrating the importance of effective pandemic response for airborne viral diseases in minimizing mortality even prior to the COVID-19 pandemics. Similarly, global death tolls for the 1918 H1N1, 1957-1958 H2N2, and 1968 H3N2 influenza pandemic were 50 million (10), 1.1 million (11), and 1 million (12), respectively, demonstrating a repeated need for effective responses that reduce morbidity and mortality during pandemics, which will continue to occur in the future.

It remains unclear what factors are associated with an effective response to infectious disease threats. Current studies assessing COVID-19 public health practices have primarily evaluated the efficacy of individual interventions, particularly focusing on the impact of masking and social distancing policies. Systematic reviews on these interventions support the benefits of physical distancing of at least one meter and masking, with stronger benefits associated with two meters distance and N95 masks (13, 14). Other inexpensive measures such as hand hygiene and cough etiquette, which have been adopted during previous influenza outbreaks, were also recommended for COVID-19 management. One past study focused on influenza showed that hand hygiene appeared to have significant protective effects, while masking provided non-significant protective effects (15). There is some evidence to support practices such as social distancing and restricting business operations (16), but these measures can be practically and economically difficult to implement (15).

Consistent policy implementation improves a range of outcomes during emergency settings (e.g., education) (17). Although there is continuing research assessing national responses to SARS-CoV-2, there are still multiple unknowns when it comes to identifying factors associated with COVID-19 responses that improve health outcomes, and more generally, any global pandemic response. Existing cross-national studies have considered only small cohorts of countries, which raises the risk of selection bias and makes it difficult to pinpoint specific interventions that reduce the spread of COVID-19. Many early studies assessed health outcomes only in the first half of 2020, prior to large outbreaks later in the year (16, 18, 19).

To fill in the gaps surrounding what constitutes an effective pandemic response, we asked two questions. First, what NPIs have been shown to be most effective in reducing morbidity and mortality during the COVID-19 pandemic prior to the availability of vaccine? Second, what are the predictors for countries' pandemic responses? The answers to these questions provide guidance on how to create public health policies during a pandemic that improve health outcomes. To answer these questions, in this study we investigated specific factors that might predict improved health outcomes during a pandemic. We examined country characteristics, extent of policy implementation, and specific non-pharmaceutical interventions (NPIs) associated with COVID-19 health outcomes, including all reporting countries through the end of 2020. We hypothesized that countries that had more comprehensive health coverage prior to the pandemic would be capable of implementing more comprehensive public health policies to prevent COVID-19 spread, resulting in better health outcomes (fewer number of cases and deaths) in 2020.

## Materials and Methods

This cross-sectional study examined associations between country characteristics, health policy interventions (both national and sub-national), and COVID-19 health outcomes across all countries that publicly reported data through December 31, 2020. The unit of analysis was countries. We compiled data from five publicly available datasets, which were downloaded between January 2021 and April 2021.

### Data Sources and Sample

1. Data for new non-pharmaceutical interventions (NPIs) implemented in response to COVID-19 were drawn from CoronaNet, an open-access dataset that collected and regularly updated data between December 31, 2019 and December 31, 2020. CoronaNet focuses on collecting information on government responses, including public health policy interventions, across 198 countries that were intended to contain the spread of the COVID-19 pandemic (20, 21). Corona indicated the number and types (mandatory and non-mandatory) of health policies implemented at different levels of government (national, subnational). Entries classified as changes to existing policies and terminations of policies were excluded. We largely categorized policies according to the CoronaNet codebook, with the following changes:

- Masking: Policies that included the word “mask” within the subcategory type name
- Social distancing: Policies classified as “social distancing” that did not contain the word “mask”
- Testing and tracing: Policies classified under “health resources,” “health monitoring,” and “health testing”
- Restriction of movement: Policies classified under “internal border restrictions,” “lockdowns,” “quarantine,” and “quarantine/lockdown”
- Border Closure: Policies classified under “external border restrictions”
- Public awareness: Policies classified as “anti-disinformation measures” and “public awareness”
- Other: Policies classified as “declaration of emergency,” “hygiene,” “new task force, bureau, or administrative configuration,” or “other policy not listed above”

2. Country characteristics data on population density, universal health coverage service index, and income-level were collected from World Bank Open Data (22).
3. Correlates of War Direct Contiguity Data provided information on number of bordering countries (23).
4. Bjoernskov-Rode Regime Data provided information on whether a country was considered a democracy or not (24).
5. Freedom House's Global Democracy Score acted as an indicator for country-level freedom, which was calculated as the sum of civil liberties and political rights scores (25).
6. WHO Coronavirus (COVID-19) Dashboard provided the number of COVID-19 cases and deaths as of December 31, 2020^1^.

All datasets are open-access and links are provided in the Study Information section. Countries with missing data were excluded from the analysis.

### Measures

The primary outcomes of the study were (a) COVID-19 policy interventions, captured as count of policies, and (b) COVID-19 health outcomes, measured as caseload per million and deaths per million at the end of 2020. We used December 31, 2020, as an endpoint anticipating that vaccine approval and distribution beginning in December 2020 would independently influence COVID-19 health outcomes in 2021. Predictors of COVID-19 policy interventions were country characteristics. Predictors of COVID-19 outcomes (cases and deaths per million) were policy counts by type. Although CoronaNet classified mask policies as a subset of other hygiene measures, given past research suggesting the importance of masking we assessed these policies separately.

### Analysis

All analysis was conducted using Stata 16 (26). We calculated descriptive statistics for country characteristics and health policy interventions. We conducted multivariate regressions to examine relationships between (a) country characteristics and the number of COVID-19 interventions by policy type and (b) the number of COVID-19 policies and COVID-19 related health outcomes.

The first part of our analysis evaluated the relationship between national characteristics with national policies; we excluded subnational policies from the analysis given that regions with countries may have different characteristics that drive policy adoption.

The second part of our analysis examined associations between COVID-19 NPIs and health outcomes. We included both national and subnational policies in this analysis on the grounds that subnational policy changes were likely to affect health outcomes. We analyzed all policies as well as conducting subgroup analyses for only those policies that were mandatory, to assess whether mandatory policies were more strongly associated with better health outcomes.

## Results

### Country and Policy Characteristics

Country characteristics and COVID-19 health outcomes are summarized in Table 1. Table 2 presents the mean and standard deviation (sd) for different policy types and average percentage of each policy type.

**Table 1:** Summary statistics for country characteristics and COVID-19 outcomes.

**Country characteristic**	**Mean**	**SD**	**Min**	**Max**	**Observations**
Number of bordering countries^a^	3.32	2.58	0	14	187
Freedom house score^b^	6.94	4.04	2	14	184
Population density^c^	303.96	1555.63	2	19348	181
UHC service coverage index^c^	64.23	15.55	25	89	174
Democracy status^d^	0.59	0.49	0	1	181
COVID cases per million (total)^e^	16,178.98	19,969.66	3	104,174	183
COVID deaths per million (total)^e^	315.68	398.64	0	1738	171
**Income category** ^**c**^	**Low**	**Low-middle**	**Middle-high**	**High**	**Observations**
Count	22	44	47	69	182

*Sources: ^a^Correlates of War Direct Contiguity Data; ^b^Freedom House's Global Democracy Score; ^c^World Bank Open Data; ^d^Bjoernskov-Rode Regime Data; ^e^WHO Coronavirus (COVID-19) Dashboard*.

**Table 2:** Summary statistics for count of policies categorized by government level, mandatory and non-mandatory.

	**National**				**National and sub-national**				**National and Sub-national**			
	**(all policies)**				**(all policies)**				**(Mandatory Policies)**			
	**Mean, min, max for countries**			**% of total policies**	**Mean, min, max for countries**			**% of total policies**	**Mean, min, max for countries**			**% of total policies**
**Policy type**	**Mean** **(SD)**	**Min**	**Max**	**Mean** **(SD)**	**Mean** **(SD)**	**Min**	**Max**	**Mean** **(SD)**	**Mean** **(SD)**	**Min**	**Max**	**Mean** **(SD)**
Masking	2.30 (3.17)	0	25	0.04 (0.04)	5.41 (14.09)	0	144	0.04 (0.04)	3.79 (10.53)	0	114	0.04 (0.04)
Social distancing	1.87 (2.41)	0	20	0.03 (0.04)	4.06 (10.94)	0	102	0.04 (0.04)	3.07 (8.42)	0	77	0.04 (0.05)
Testing and tracing	13.86 (22.88)	0	262	0.20 (0.11)	28.82 (82.13)	0	842	0.20 (0.10)	18.94 (59.36)	0	688	0.17 (0.11)
Border closure	8.20 (8.71)	0	60	0.15 (0.11)	9.91 (14.96)	0	169	0.13 (0.10)	8.28 (10.98)	0	119	0.15 (0.11)
School closure	2.81 (3.35)	0	23	0.05 (0.04)	7.76 (24.81)	0	261	0.06 (0.04)	6.88 (21.30)	0	210	0.07 (0.04)
Restriction of movement	8.02 (8.91)	0	64	0.14 (0.08)	18.66 (49.32)	0	579	0.15 (0.09)	16.51 (41.95)	0	492	0.18 (0.10)
Public awareness	5.59 (9.48)	0	96	0.08 (0.07)	10.69 (29.06)	0	275	0.08 (0.06)	4.25 (13.23)	0	140	0.04 (0.04)
Restriction on gatherings	8.34 (9.65)	0	90	0.15 (0.09)	21.49 (61.19)	0	597	0.16 (0.09)	19.48 (54.91)	0	537	0.18 (0.11)
Other policies	9.19 (10.25)	0	66	0.15 (0.10)	17.72 (49.45)	0	495	0.15 (0.09)	13.20 (39.62)	0	380	0.14 (0.10)

*Source: CoronaNet; N = 198*.

At the national level, the least common policies implemented across all countries (*n* = 198) were related to social distancing (1.87; sd 2.41), and the most common were testing and tracing policies (13.86; sd 22.88). Results were comparable for data that considered both national and subnational policies. Countries implemented an average number of 2.30 (sd 3.17) masking policies on the national level and 5.41 (sd 14.09) policies on both the national and subnational levels, with 3.79 (sd 10.53) of these being mandatory. Countries implemented an average number of 1.87 (sd 2.41) social distancing policies on the national level and 4.06 (sd 10.94) policies when considering both national and subnational levels, with 3.07 (sd 8.42) of these being mandatory. Masking policies and social distancing policies each accounted for a mean of 0.04% (sd 0.04) of total policies at the national level and all government levels, including the subset of only mandatory policies (Table 2).

### Country Characteristics as Predictors for Policy Implementation

Table 3 presents findings of multivariate regression analyses assessing the associations between country characteristics and the types of national policies implemented. The count of bordering countries was positively associated with the number of masking (0.24, *p* < 0.05), border closure (0.78, *p* < 0.01), and school closure policies (0.39, *p* < 0.01). An increase in one unit on the Universal Health Coverage Service Index was associated with an increased number of policies for masking (0.06, *p* < 0.05), border closure (0.16, *p* < 0.05), public gatherings (0.20, *p* < 0.05), and other policies (0.23, *p* < 0.01). Higher population density was associated with a higher count of all masking (0.001, *p* < 0.01) and school closure policies (0.002, *p* < 0.01).

**Table 3:** Multivariate regressions for associations between country characteristics and policy implementation by government level (for all national policies).

**National (all policies)**									
**Variable**	**Masking**	**Social distancing**	**Testing and tracing**	**Border closure**	**School closure**	**Restriction of movement**	**Restrictions on gatherings**	**Public awareness**	**Other policies**
Number of bordering countries^a^	0.24* (0.10)	0.09 (0.09)	0.86 (0.82)	0.78** (0.28)	0.39** (0.11)	0.57 (0.31)	0.11 (0.34)	0.43 (0.34)	0.39 (0.34)
Freedom score^b^	−0.05 (0.11)	−0.12 (0.09)	0.55 (0.88)	0.19 (0.30)	0.013 (0.12)	−0.08 (0.33)	0.12 (0.36)	0.05 (0.37)	0.87* (0.37)
UHC service index^c^	0.06* (0.02)	0.04* (0.02)	0.37* (0.19)	0.16* (0.07)	0.04 (0.03)	0.09 (0.07)	0.20* (0.08)	0.13 (0.08)	0.23** (0.08)
Democracy^d^	−1.11 (0.78)	−0.15 (0.64)	−1.36 (6.14)	0.79 (2.10)	−0.74 (0.83)	−1.71 (2.30)	−3.45 (2.53)	0.07 (2.56)	−0.75 (2.57)
Population density^c^	0.001** (0.00)	0.000 (0.00)	0.001 (0.00)	0.002 (0.00)	0.002 (0.00)	0.002 (0.00)	0.000 (0.00)	0.001 (0.00)	0.001 (0.00)
Income categories^c^ (reference: low income)	-	-	-	-	-	-	-		-
Low-middle income	−0.28 (0.87)	0.11 (0.73)	1.29 (7.03)	2.98 (2.40)	1.53 (0.95)	3.08 (2.63)	0.91 (2.90)	1.67 (2.93)	3.08 (2.94)
Upper-middle income	−1.94 (1.00)	0.13 (0.84)	−0.49 (8.12)	0.58 (2.78)	−0.23 (1.10)	3.14 (3.04)	−1.06 (3.35)	−0.11 (3.39)	0.41 (3.40)
High income	−0.94 (1.15)	0.51 (0.94)	2.56 (9.07)	3.92 (3.10)	0.89 (1.223)	5.03 (3.40)	0.65 (3.74)	1.81 (3.78)	2.40 (3.79)
Constant	−0.96 (1.88)	−0.15 (1.57)	−15.90 (15.20)	−9.62 (5.19)	−1.54 (2.05)	−1.27 (5.68)	−3.45 (6.26)	−9.46 (6.33)	−14.20* (6.34)
Observations	167	167	167	167	167	167	167	167	167
R^2^	0.17	0.14	0.06	0.18	0.20	0.12	0.10	0.07	0.17

Standard errors in parentheses *p < 0.05, **p < 0.01.

*Sources: ^a^Correlates of War Direct Contiguity Data; ^b^Freedom House's Global Democracy Score; ^c^World Bank Open Data; ^d^Bjoernskov-Rode Regime Data*.

*Dashboard; all policy data drawn from CoronaNet*.

We expected that national characteristics might be poor predictors of subnational policies, so we conducted a sensitivity analysis with the same multivariate regressions for policies at all government levels. We found that only the number of bordering countries was associated with a change in the number of policies, and the same was true for mandatory policies at all government levels.

### Policies as Predictors for COVID-19 Outcomes

Table 4 presents findings from the regression analysis analyzing associations between the count of policies in different categories and COVID-19 health outcomes. We included all policies, both national and subnational, in this analysis, anticipating that even subnational interventions could be associated with changes in overall caseloads and deaths. We then considered mandatory policies alone to assess whether mandatory changes had a stronger influence.

**Table 4:** Multivariate regression for associations between select policies and COVID-19 health outcomes.

**National and sub-national (all policies)**								
**Predictor**	**COVID-19 cases per million** ^**b**^	**COVID-19 deaths per million** ^**b**^	**Predictor**	**COVID-19 cases per million** ^**b**^	**COVID-19 deaths per million** ^**b**^	**Predictor**	**COVID-19 cases per million** ^**b**^	**COVID-19 deaths per million** ^**b**^
Count of social distancing policies^a^	909.30** (2.99)	15.33* (2.50)	Count of social distancing policies	1071.70*** (3.49)	23.39*** (3.84)	Count of social distancing policies	711.70** (3.13)	14.35** (3.16)
Count of masking policies^a^	−496.10* (−2.10)	−7.57 (−1.59)	Count of testing and tracing policies	−108.50** (−2.65)	−2.47** (−3.04)	Count of restriction of movement policies	−102.30* (−2.03)	−2.10* (−2.09)
Constant	15034.90*** (9.89)	289.30*** (9.09)	Constant	14813.90*** (9.86)	286.90*** (9.25)	Constant	15051.30*** (9.88)	291.40*** (9.20)
Observations	188	174	Observations	188	174	Observations	188	174
R^2^	0.06	0.05	R^2^	0.07	0.09	R^2^	0.06	0.06
**National and sub-national (Mandatory policies)**								
Count of social distancing policies^a^	1393.40*** (3.48)	26.70** (3.32)	Count of social distancing policies	1116.90*** (3.67)	24.41*** (4.05)	Count of social distancing policies	915.70** (3.20)	19.46*** (3.42)
Count of masking policies^a^	−867.60** (−2.62)	−16.17* (−2.43)	Count of testing and tracing policies	−116.50** (−2.66)	−2.66** (−3.06)	Count of restriction of movement policies	−119.80* (−2.06)	−2.64* (−2.28)
Constant	15012.30*** (9.80)	291.90*** (9.20)	Constant	14796.80*** (9.69)	287.20*** (9.18)	Constant	15221.00*** (9.78)	296.70*** (9.25)
Observations	183	171	Observations	183	171	Observations	183	171
R^2^	0.07	0.07	R^2^	0.07	0.09	R^2^	0.06	0.07

t statistics in parentheses, *p < 0.05, **p < 0.01, ***p < 0.001.

*N = 198*.

*Sources: ^a^CoronaNet; ^b^WHO Coronavirus (COVID-19) Dashboard*.

Given the small size of the population (*N* = 198 countries), we did not anticipate that the analysis could support multiple predictors. We first conducted bivariate regressions for all six policy categories as well as paired policy combinations. In the bivariate regression analyses, only social distancing policies were significantly associated with COVID-19 health outcomes.

We proceeded by conducting multivariate regressions for each of the remaining policy categories combined with social distancing policies (mandatory and non-mandatory). When social distancing policies were coupled with masking, testing and tracing, or restriction of movement policies, social distancing policies were consistently associated with a positive increase in both COVID-19 cases per million and deaths per million. Masking policies were associated with a decrease in COVID-19 cases per million (−496.10, *p* < 0.05), and testing and tracing policies were associated with a decrease in COVID-19 cases per million (−108.50, *p* < 0.01) and deaths per million (−2.47, *p* < 0.01). Similarly, restriction of movement policies were associated with a decrease in COVID-19 cases per million (−102.30, *p* < 0.05) and deaths per million (−2.10, *p* < 0.05).

Our analysis of mandatory policies only found that social distancing policies were associated with an increase in both COVID-19 cases per million (1393.40, *p* < 0.001) and deaths per million (26.70, *p* < 0.01), while masking policies were associated with a decrease in COVID-19 cases per million (−867.60, *p* < 0.01) and COVID-19 deaths per million (−16.17, *p* < 0.05). Testing and tracing policies were associated with a decrease in COVID-19 cases per million (−116.50, *p* < 0.01) and deaths per million (−2.66, *p* < 0.01). Restriction of movement policies were associated with a decrease in COVID-19 cases per million (−119.80, *p* < 0.05) and deaths per million (−2.64, *p* < 0.05).

## Discussion

### Summary of the Evidence

Although there is little empirical literature examining associations between public health interventions and COVID-19 outcomes cross-nationally, our results are generally consistent with the findings of existing studies. We identified specific NPIs associated with COVID-19 health outcomes globally, as well as country characteristics that predicted the implementation of these interventions. The strongest predictor for frequency of policy implementation was the number of bordering countries. Countries with a higher number of neighboring countries may be prompted to address a pandemic spread due to a higher likelihood of international travel and population mixing. This is in contrast to countries with few or no bordering countries, for which geography poses an inherent barrier to international travel. In addition, we found that countries with higher population density were more likely to implement masking policies, potentially reflecting the need to institute tighter COVID-19 infection controls in crowded areas. This aligns with current studies suggesting the spread of COVID-19 in some countries may have been associated with certain cities or districts that have higher population density (27, 28). This expectation may also explain why high population density is a country characteristic associated with increased school closures. A study by Haug et al. reported similar findings. They ranked the effectiveness of world-wide government interventions by quantifying policies with the reproduction number of COVID-19 and concluded that while no single intervention can reduce the spread of COVID-19, NPIs such as canceling small gatherings, school closures, restrictions on movement, border restrictions, and national lockdowns helped reduce transmission (29). Conversely, an exploratory analysis of 50 countries using data through April 2020 concluded that lockdowns and wide-spread testing were not significantly associated with COVID-19 mortality; however, this study was conducted prior to the waves of large outbreaks in 2020 (30).

Higher levels of universal health coverage were associated with more policies relating to masking, social distancing, testing and tracing, border closures, and public gatherings. This pattern suggests that countries that have committed to provide care for residents may have a greater incentive to prevent the spread of infectious disease or have the necessary infrastructure to implement extensive NPIs. Among democratic countries, it provides support for the concept of “patient-citizens,” where higher service coverage translates to more citizens funding health services and consciously voting for and holding political representatives responsible for shaping health policies (31). While our study did not directly examine the association between higher levels of universal health coverage and COVID-19 health outcomes, the inadequate response to the COVID-19 pandemic by countries ranked highly on the GHS index indicates there are other factors which may play a role in COVID-19 health outcomes and are beyond the scope of this paper, such as political violence, partisan protests, and government leadership.

The government's ability to enforce mandatory policies may also influence the effectiveness of a country's pandemic response. Consistent with previous research, we found that masking policies were associated with better COVID-19 health outcomes, and this relationship was particularly strong when policies were mandatory. This is consistent with an interrupted time-series analysis of six Latin American countries, which found that mandatory quarantines slowed case rate growth in some countries but not others, and that mandatory mask policies appeared to reduce the spread of COVID-19 (18). This result parallels our findings that mandatory mask policies were associated with reduced COVID-19 cases and deaths per million. Masking policies also have the potential to reduce the spread of other infectious diseases with respiratory modes of transmission such as influenza, and avoiding delayed management of other diseases can prevent indirect COVID-19 deaths or long-term COVID-19 complications (13, 14). Similarly, both testing and tracing policies and restriction of movement policies were associated with improved COVID-19 health outcomes, with more reductions in cases and deaths for mandatory policies. Our findings for restriction of movement policies are consistent with previous research demonstrating that lockdowns and closures of businesses are effective in decreasing infection rates and COVID-19 deaths (32, 33). However, current literature has not attempted to compare the benefits of restricted movement to those anticipated with NPIs such as lockdowns or business closures (34). There is limited evidence based on real-world data assessing the effectiveness of testing and tracing policies, but our findings are consistent with modeling studies that suggest that these policies may result in reduced time in quarantine and reduced disease spread (35–37).

Unexpectedly, we found that the count of social distancing policies was associated with increased COVID-19 deaths and cases per million in both the “mandatory” and “all policies” analysis. This effect persisted through multiple sensitivity analyses using different combinations of country characteristics and policy types. We suspect that this effect is not due to social distancing policies themselves but may be a reflection of policy implementation and a misunderstanding of the effects of social distancing on the risk of infection. Countries may have continued to implement additional social distancing policies that attempted to increase safety for people spending long periods of time with others in unventilated, indoor spaces. We only evaluated the impact of the implementation of new policies on the spread of COVID-19 but did not evaluate changes to implemented policies. Countries that continuously modify and improve their implemented policies may yield better health outcomes. The effectiveness of social distancing depends on the setting, and it appears to be less effective indoors than in outdoor areas or in buildings with better ventilation (38). Most of the social distancing policies identified by CoronaNet did not attempt to assess these factors. In addition, maintenance of distances at the lengths presumed to be effective in preventing the spread of COVID-19 may be difficult to enforce and monitor compared to an intervention such as masking, where adherence is immediately apparent.

Our findings are largely congruent with current predictive mathematical modeling on the effects of NPIs—with the only exception being the impact of social distancing policies. As such, our study, which leveraged real-world data, provides critical evidence supporting these modeling predictions that suggests their validity. Our findings for restriction of movement interventions, including quarantine and lockdown interventions, generally support the findings from Ferguson et al. (39), which simulated the effects NPIs could have had on infection spread from January 2020 to March 2020 in the United States and the United Kingdom using early data from Wuhan, China. One notable incongruency is on the prediction for the effect of social distancing on preventing infection. We suspect that this may be due to variations in the nature of social distancing policies. Our findings also provide support for the SIDARTHE model proposed by Giordano et al. (40), which delineated between severities of illness and detected vs. undetected cases of infection. Consistent with our findings, this model predicted a decreased reproduction number in Italy translating to decreased deaths and COVID-19 infections, resulting from stricter lockdowns and expanded testing and tracing policies. As with Ferguson's model, the SIDARTHE model predicts benefits from social distancing in theory, however our results indicate that their real-world implementation may not have the anticipated effects. Modeling studies from single countries, including India, are also consistent with our global findings, as they predicted decreases in disease transmission due to lockdown (41) and masking (42). However, modeling of public awareness measures as an additional intervention predicted these would contribute to reducing COVID-19 spread in India (43) while our study of multiple countries did not identify improvements in COVID-19 outcomes for interventions related to public awareness measures.

### The Emerging Situation in the Health Sector

Although pharmaceutical interventions, including the COVID-19 vaccine, remdesivir, and dexamethasone, have shown clinical benefits, these interventions take time to research, develop, and implement (44, 45). Moreover, the SARS-CoV-2 virus has rapidly mutated throughout the pandemic, exhibiting varying degrees of transmission rates and disease severity (46). Our study focuses on the real-world effectiveness of NPIs prior to the widespread availability of COVID-19 vaccines, but the continued importance of these NPIs in the context of vaccination and COVID-19 therapeutics is demonstrated by continued difficulties in identifying and distributing effective pharmacologic therapies. As the complex dynamic between emerging variants, vaccine efficacy, and effective pharmaceuticals continues to evolve during an ongoing COVID-19 pandemic, efforts to identify NPIs that reduce disease transmission will remain critical while therapeutic interventions to prevent and treat disease spread are not yet established and accessible worldwide.

### Strengths and Limitations

This study has limitations. The analysis of COVID-19 interventions relied on publicly accessible, secondary data compiled by CoronaNet, which may not comprehensively catalog all interventions from certain countries, such as low- and middle-income countries that may lack support for reporting infrastructure. To compare interventions, we selected and analyzed specific policy types that were commonly implemented based on available evidence. Factors such as cultural beliefs surrounding viral transmission and protests against COVID-19 policies were not available or well-defined, even though they may have affected COVID-19 health outcomes. Our study design could not account for adherence to guidelines because evaluation of adherence at each country was not systematic at the time of analysis. Our multivariate regression analysis only included new policies that were implemented and could not evaluate the association between updated policies and COVID-19 health outcomes. Finally, due to the cross-sectional nature of the study, COVID-19 outcomes at the chosen timepoint might not reflect contemporaneous COVID-19 morbidity or mortality. Despite these limitations, the findings in this cross-national analysis suggest NPIs that could guide future pandemic response as well as ways to identify countries that may be more likely to implement them.

### Recommendations for Research

Analyzing similar data over a more extended period could provide a more comprehensive understanding of relationships between public health interventions and health outcomes. For example, the spread of new SARS-CoV-2 variants with different characteristics and transmissibility, in combination with the distribution of vaccines, have changed national outcomes since 2020. Our study considered only policy counts; future studies could build on these findings by evaluating the comprehensiveness and quality of the implemented policies. Identifying and including measures of population adherence could also provide a more detailed understanding of the relationships between NPIs and health outcomes. Questions have been raised regarding how long to continue NPI policies during vaccination rollouts, given that these policies are restrictive and sometimes unpopular (47, 48). As such, continuing research assessing the length of time to utilize NPIs from a policy standpoint is important.

## Conclusions

Our research contributes to a growing base of knowledge about public health policy interventions that can prevent the spread of COVID-19. We provide novel information on factors that can predict countries' pandemic preparedness by evaluating the associations between country characteristics, policy implementation, and health outcomes. Our findings suggest that NPIs, such as masking and testing and tracing policies, can prevent or delay disease cases and deaths, giving researchers time to develop pharmaceutical interventions. Additionally, our research supports modeling studies and a growing base of evidence emphasizing the importance of masking in limiting the spread of infectious diseases. Our counterintuitive finding on the relationship between the creation of more social distancing policies and worse COVID-19 health outcomes suggests that countries should be cautious in implementing them and continue to assess potential unintended effects.

## Data Availability Statement

The data supporting the conclusions of this article are publicly available at the following websites:

1. CoronaNet Database Version 1.0 (core): https://www.coronanet-project.org/download.html.
2. World Bank Open Data: https://data.worldbank.org/.
3. Correlates of War Direct Contiguity Data v3.2: https://correlatesofwar.org/data-sets/direct-contiguity.
4. Bjoernskov-Rode Regime Data v3.2: http://www.christianbjoernskov.com/bjoernskovrodedata/.
5. Freedom House's Global Democracy Score: https://freedomhouse.org/countries/freedom-world/scores (data download available by request from Freedom House).
6. WHO Coronavirus (COVID-19) Dashboard: https://covid19.who.int/info/.

## Author Contributions

AC, AH, JL, and NT all contributed equally to the study conception, design, data collection, data analysis, manuscript preparation, and revisions of this research project. TL and DA supervised the project in addition to assisting with Stata 16 coding, manuscript revisions, design, and data analysis. All authors contributed to the article and approved the submitted version.

## Conflict of Interest

The authors declare that the research was conducted in the absence of any commercial or financial relationships that could be construed as a potential conflict of interest.

## Publisher's Note

All claims expressed in this article are solely those of the authors and do not necessarily represent those of their affiliated organizations, or those of the publisher, the editors and the reviewers. Any product that may be evaluated in this article, or claim that may be made by its manufacturer, is not guaranteed or endorsed by the publisher.

## Footnotes

^1^Available online at: https://covid19.who.int/info/ (accessed May 5, 2021).
